# A formative study to understand perspectives of families eligible for a pediatric obesity program: a qualitative study

**DOI:** 10.1186/s12889-018-5466-x

**Published:** 2018-05-02

**Authors:** Rachel G. Tabak, Nishita Dsouza, Cynthia D. Schwarz, Karyn Quinn, Patricia Kristen, Debra Haire-Joshu

**Affiliations:** 1 The Brown School, Washington University in St. Louis, 1 Brookings Dr, St. Louis, MO 63130 USA; 2 Envolve PeopleCare, 20 Batterson Park Road, Farmington, CT 06032 USA; 3 The Brown School and The School of Medicine, Washington University in St. Louis, 1 Brookings Dr, St. Louis, MO 63130 USA

**Keywords:** Childhood obesity prevention/treatment, Qualitative formative evaluation, Low-income population, Health coaching

## Abstract

**Background:**

Raising Well^®^ (RW) was initiated in 2015 by Envolve PeopleCare™ at the request of health plans seeking a solution to work with families on Medicaid that have a child with overweight or obesity. RW uses expert clinical coaches via phone contact to deliver an educational intervention promoting lifestyle change to families with at least one overweight or obese child in an eligible Medicaid health plan. This gives RW significant potential for reach and population impact. This project aimed to understand how to maximize this impact by exploring perspectives of RW, using a conceptual framework informed by the Conceptual Model of Implementation Research, including assessment of the feasibility, acceptability, and appropriateness of RW; determining satisfaction among those experiencing coaching; identifying reasons individuals do not participate; and developing recommendations to enhance interest and participation.

**Methods:**

Semi-structured interviews were conducted with 70 RW-eligible families across four states, who were described as: active participants, respondents who dropped or stopped RW, and RW non-participants. Following the interviews, the transcripts were coded inductively and deductively using a grounded theory approach, considering themes from the conceptual framework; themes also emerged from the data.

**Results:**

From this sample, 19 families reported to be active coaching participants, 24 had dropped coaching, and 27 were RW non-participants. A number of themes were identified. Feasibility themes included coaches’ flexibility and willingness to work with the family’s schedule. Acceptability themes suggest providing actionable strategies tailored to the family’s context and needs, beyond just nutrition information and tips, early in the coaching relationship so the family perceives a benefit for continued participation. With regard to appropriateness, families were also interested in other methods of communication including email, texting, and in person visits. Access to resources for activity and healthy eating in their local community was also recommended.

**Conclusions:**

RW has the potential to improve health and promote wellness. To enhance the impact of this program, RW could incorporate these findings to promote feasibility, acceptability, and appropriateness and improve program implementation. Strategies may include modifying the information provided or the mode of delivering the information.

## Background

Obesity is prevalent among children and adolescents in the United States [1–5]. Excess adiposity adversely affects children during childhood [6–14], and can predispose children to chronic health conditions when they become adults [6, 7, 15, 16]. This is particularly true for children from low-income families, such as those served by Medicaid (a safety net health insurance program in the United States) [17–20]. Interventions to promote healthy eating and activity behaviors, which can be disseminated and implemented widely, are needed to enhance reach and sustainability. To bridge this gap between research and practice, an industry-academic partnership was developed to bring the best available evidence on behavior change into real-world practice [21, 22].

The industry partner, Envolve PeopleCare™, launched the Raising Well^®^ (RW) program in 2015 at the request of health plans seeking a solution to work with families on Medicaid that have a child with overweight or obesity. Envolve PeopleCare is a subsidiary of Centene Corporation, one of the largest Medicaid Managed Care providers, via state health plans, in the US. RW is currently offered in seven states and provides telephonic health coaching with a dietitian or exercise specialist to help caregivers improve the nutrition and activity behaviors of the child with overweight or obesity. RW works with parents to create a supportive home environment where healthy lifestyle changes can occur, and is based on motivational interviewing, and therefore is driven by participant goal selection. Many states mandate that the health plans address pediatric obesity. This gives RW significant potential for reach and population impact as the program is gradually being expanded to more states.

Implementation science explores not only the intervention (in this case, RW), but also the implementation strategies used to put the intervention in place within a practice setting (in this case, Envolve PeopleCare) [23]. Furthermore, implementation science distinguishes between intervention outcomes (e.g., child health behaviors) and other important outcomes. Proctor et al. lay out a model, which defines implementation outcomes as the “effects of deliberate and purposive actions to implement new treatments, practices, and services” [24]. These include service outcomes (e.g., number of RW calls completed) and implementation outcomes (e.g., acceptability, feasibility, and appropriateness of RW) [25]. Feasibility can be defined as the extent to which RW fits well and is useful for eligible families, and the extent to which they are able to carry out the program; acceptability as the perception that RW is agreeable, palatable, or satisfactory; and appropriateness as fit, relevance, or compatibility of RW for eligible families. As described in the model by Proctor et al., implementation outcomes function as indicators of the implementation success and as indicators of implementation processes. To improve practice and inform future interventions with this population, the academic partners developed a formative research study guided by implementation science [26].

The goal of this formative study was to evaluate the intervention (RW) and the aspects of the multicomponent implementation strategy in terms of three implementation outcomes: acceptability, feasibility, and appropriateness [25].

## Methods

The study population included the caregivers of children eligible for the RW pediatric obesity program in four states (Florida, Louisiana, Missouri, and New Hampshire). To be eligible for RW, a child must be enrolled in an included health plan, be between 2 and 18 years old, and be overweight or obese (body mass index (BMI) ≥85th percentile). Purposive sampling was used so that participants represented different levels of engagement with RW based on administrative data: 1) current coaching families, 2) families who dropped coaching, 3) families who declined coaching, and 4) those who were not able to be contacted by a health coach. In Florida, families that could not be reached by the RW coaches (group 4) were not recruited for the current study, as data saturation with group 4 respondents had already been reached. In addition, Missouri had only recently started offering RW at the time of this study, and therefore only a small number of MO members were eligible for this study; only members listed as current participants in RW in Missouri were recruited. Upon speaking with respondents during their interviews, it became apparent that the administrative data categorization for the respondent’s engagement level in RW differed from the interview participant’s own perspective of their level of engagement, so respondents were organized into three groups based on their reported engagement (see Table 1). The first group, active participants, included those who were currently enrolled in RW and those who had been previously enrolled and completed the program (RW participants). Those who had initiated health coaching and subsequently dropped the program or stopped participating in RW (e.g., stopped answering calls) were the second group (dropped). Non-participants included those who declined to participate in RW, those who reported not receiving any communication about the program, and those who were not able to recall whether they had participated in RW (RW non-participants).

**Table 1:** Participants, dropped, and non-participants of interviewees in the RW program across states

State	Eligible participants	Members contacted	Total interviewed	RW participants	Dropped RW	RW non-participants
Louisiana	434	99	15	5	5	5
Missouri	23	14	6	4	2	0
Florida	392	55	21	2	9	10
New Hampshire	197	179	28	8	8	12
Total	1046	347	70	19	24	27

Eligible families were sent an outreach mailing that included a recruitment flyer. Following the outreach mailing, interviewees were contacted via telephone by trained interviewers; verbal consent was obtained before beginning the interview. Calls were conducted between March 2016 and December 2016. Interviews were conducted in Spanish and English. Participants were encouraged to speak openly, and reassured that Washington University in St. Louis was a third-party unaffiliated with the RW program and that interviewee responses would not affect the status of a family in the RW program or their insurance status. Participants received a $25 gift card for their time. The study protocol was approved by the Human Research Protection Office at Washington University in St. Louis.

The semi-structured interview guide was developed using the implementation framework developed by Procter et al. [25], and included open-ended questions and probes to assess: program perceptions; knowledge gained and goal-setting within the program; behavior change resulting from RW; whether or not the respondent would recommend the program to others; and recommendations regarding changes to the program. Participants were also invited to share any specific concerns they had with the program. Data saturation was determined when new data collected was repeating perspectives expressed previously, with no new data emerging [27, 28].

Phone interviews were recorded for transcription purposes. Interview transcripts were imported into NVivo 10 and coded using deductive focused coding techniques [28]. A draft code book was developed from the interview guide and informed by the implementation outcomes framework developed by Proctor et al. [25] Feasibility, acceptability, and appropriateness were selected as the relevant implementation outcomes. The research team then coded several interviews to observe the appropriateness of initial codes and to incorporate emerging themes. The code book was iterated twice. Data in the interview transcripts were coded for positive, negative, and/or neutral responses such that positive, negative, and/or neutral perspectives on a theme were all included. All interviews were double coded. Further refinement of the themes was completed after all interviews had been coded. All qualitative analyses were conducted in NVivo 10 and Microsoft Excel.

## Results

There were 70 interviews conducted across the four states; the number of eligible members, members contacted, and members per group and per state are summarized in Table 1. The active participants group (RW participants), dropped or stopped RW group (dropped), and the group of non-participants (RW non-participants) included 19, 24, and 27 respondents, respectively. Of the 21 interviews that were conducted in Florida, 12 were in Spanish; the remainder of the interviews were conducted in English. The average length of an interview was 8.72 min.

Table 2 shows the themes within the implementation outcomes as well as the number of participants, dropped, and non-participants that discussed these themes and illustrative quotes.

**Table 2:** Implementation outcomes identified among RW participants, dropped, and non-participants

Implementation outcome	Theme	Quote	Participants	Dropped RW	Non-participants
Feasibility	Call Scheduling	“She was very accommodating too, coz usually I’m not home during the day. I’m home… like at night. I’m just on vacation this week from school, so…”	8	12	3
	Scheduling accommodations	“Um, I only talked to them twice, because they never called when they were supposed to.”	8	12	2
	Call Frequency	“no they weren’t over bearing but they didn’t call too much or not enough. I think they spread it nicely, so I think didn’t feel like I was overwhelmed.”	6	6	3
Acceptability	Content/ Advice	“Oh the dietitian that called and she wanted to you know see if um, it was a while ago, it was at least six months ago and you know and she just basically wanted to see if I wanted to discuss you know any, if I needed any advice on what I’m feeding my daughter basically nutrition,”	14	22	5
	Goal-setting	“I can remember making goals, but I can’t remember exactly. I think one of the goals was uh drinking more water for both of us and… which we have done, I mean that’s all we drink in between. I have coffee in the morning and she sometimes will have a coffee in the morning, but most of the time she’s straight up water all day.”	11	4	2
	Making Changes	“Yep, yeah, he cut down… – We’re down to I think it was, two…we’re down to like two sugary drinks a week, … and eating out, that’s like once every two weeks. So… that’s, y’know, it kinda made him aware as well, too.”	12	9	1
	Tailored recommendations	“Yeah, I think, … especially during this... that first call... kinda got to know, like, what... how… when and what times we eat, and what Tony’s schedule is… even at school. Um, what times he eats at school. And then, also just activities and when are... kind of, his weekly schedule is. So, I think it would be helpful for her to give more advice of, maybe how to implement more things. That would be (takes a breath in) … easy and not hard on our schedules, at least to…”	11	13	2
	Coach Relationship	“She’s actually been a wonderful support system.”	11	14	2
	Providing cues to action	“That is really, yeah that’s also good, it gives, sometimes it’s used as a reminder, you know like a reminder or like you know like are you doing this, are you following up on this.”	5	3	1
	Resources/ Referrals	“no, I think she was really good, coz she also offered… you know, if we needed to talk to … what was like it… a exercise or fitness person that they have, too. …you know, so she offered some other things, too.”	12	6	5
	Mailed resources	“Yes, she offered to have other people to contact me, and I said no. She offered to send packages out, and um to give us ideas of what to feed her, exercise events, but… well, yeah.”	12	6	4
Appropriateness	Constraints/ Reach	“We, we go through some crises here. So, sometimes I just can’t...take a call.”	9	12	17

### Feasibility

The implementation outcome feasibility included the themes call scheduling and call frequency, with similar frequency of discussion between participants and non-participants. RW participants agreed that a program benefit was a coach that accommodated the family’s schedule (e.g., evening calls). However, families found it difficult to engage in RW if they felt their coach did not call at the scheduled time, missed scheduled calls, or called at inconvenient times (e.g., during dinner, while they were at the store, while they were at work); this was more common among respondents who dropped or stopped RW. With regard to call frequency, bi-weekly calls were preferred.

### Acceptability

The majority of the themes were related to the outcome acceptability. These included: content/tailoring/goals, making changes, providing cues to action, coaching relationship, and resources/referral(s). RW participants found coaching content such as actionable strategies, tailored to their family and its context, to be most useful, as opposed to standard nutrition information, which was regarded as redundant. An important issue related to the content of RW was the tailoring of the recommendations provided by the health coach to the family and the family’s context (e.g., financial situation, cultural context, neighborhood built environment, environment within the home). RW participants who described a positive experience with RW felt the program was tailored to their family and were able to provide specific information they had learned, articulate goals they had set, and describe examples of changes they made from being in the program. Those who had dropped RW reported they had not set goals or could not remember the goals they had set. Behavioral changes reported included drinking more water and paying attention to portion size. Even when they did not feel the information provided by the coach was particularly useful or relevant, some RW participants felt that the coaching calls and the regular engagement of talking with a health coach served as a cue to action, supporting their behavior change efforts. Reporting that information was not tailored or was redundant was more common among respondents who dropped or stopped RW.

RW participants did appreciate when coaches were able to provide referrals. A benefit of the RW program nested within the Envolve PeopleCare organization is access to other professionals, such as an exercise specialist, as member needs become apparent. In addition to referrals, there was a positive response by some respondents to educational materials the coaches were able to send by mail. Respondents thought it would be helpful to receive materials, particularly child-friendly recipes. It is important to note that while respondents mention alternate communication channels (e.g., mail, email, website, smartphone applications), they only expressed interest in the channel the family already used, and were not interested in using a new channel. This suggests channels offered to families be flexible and tailored to the family’s communication preferences.

Overall, RW participants valued supportive, caring relationships with their coach. Some of the coaching interaction required significant assessment from the participants, and if this was perceived as excessive, it could interfere with the coaching relationship (i.e., if respondents perceived the interaction to be more like a list of questions, than a program for their child), whereas efforts on the part of the coach to get to know the family were greatly appreciated. Another aspect of the coaching relationship participants appreciated was when the coach engaged directly with the child, so the respondent did not feel like a mediator.

### Appropriateness

Within appropriates, constraints/reach was an important theme. The families participating in RW had many stressors in their lives (e.g., financial issues, childcare), which took precedence over healthy eating and activity. Part of tailoring RW to families requires awareness of these important issues so they can be taken into account and working with the family where they are.

## Discussion

This evaluation of a childhood obesity program with families enrolled in Medicaid, which focused on feasibility, acceptability, and appropriateness provided important insights. Respondents stated they wanted information tailored to their family and their family’s context, which goes beyond generic nutrition information [29]. As the RW program is based on motivational interviewing, goal setting is an important component of the program; however, as identified in the acceptability themes, many respondents who dropped or stopped RW were not able to articulate the goals they set as part of RW. It is possible they did not set goals in the program or the goals were not particularly salient to the family, perhaps related to appropriateness for families with significant barriers related to social determinants of health [30, 31]. Attention to participant goal selection is also supported by research suggesting the benefits of intrinsic motivation, underpinned by Self-determination Theory [32]. Intrinsic motivation is contrasted with extrinsic motivators such as monetary or material incentives. Selecting goals which are of importance to the participant can build intrinsic motivation, and perhaps prevent dropout, as RW participants who described a positive experience with RW were able to articulate goals they had set as part of RW. Other intervention studies and reviews have found that family-based approaches and use of tailoring can lead to greater benefits in childhood obesity prevention and treatment [33–40]. This is particularly important for families with low income [17], and is supported by other studies looking at parent perspectives [41].

Scheduling issues and the ability to incorporate multiple family members also arose as important feasibility and appropriateness findings. The families served by RW face a number of challenges in terms of access to communication, which can include changing residence (and phone numbers), issues with paying cell phone bills (which can lead to the phone service being cut off until the bill is paid), and reaching the maximum number of minutes allowed for use in a month before the month has ended; these challenges can make it hard to stay in touch with and serve this population. Attention to such implementation factors can improve future intervention efforts and the overall program expertise for future participants [25, 26, 42–44].

Positive aspects of the current RW program can be emphasized while aspects perceived less positively by those who dropped or stopped RW (e.g., issues with scheduling, content perceived as redundant, limiting questions to those required to understand the family context) can be refined to improve the overall program experience. Further, understanding respondent perspectives of the implementation outcomes can be informative for those seeking to implement obesity prevention and treatment interventions or to work with families like those eligible for RW.

This study has limitations worth noting. While the respondents were dispersed across the country, only four states were represented. This study also included only members eligible for the RW program, but did not include the perspectives of the health coaches delivering RW. Further, though some respondents were included in the current study that RW coaches had not been able to contact, this was a small number of participants, suggesting the perspectives of this population may be underrepresented. However, data saturation was reached, in this group (i.e., those RW coaches were unable to contact), when outreach to this group was stopped. This study is also limited in that it only solicits the perspectives of respondents, to identify potential changes to the program, but does not test alternate interventions, to determine whether these changes might enhance program uptake, adherence, and/or effectiveness; future research should evaluate these factors.

## Conclusions

This study provides valuable perspectives from families eligible for an obesity prevention/treatment program for children enrolled in Medicaid. These perspectives address the feasibility, acceptability, and appropriates of the program. Future research could incorporate these perspectives to interventions in this population to determine if program perceptions and program efficacy are improved.

## Abbreviations

BMI: Body mass index
RW: Raising well
